# Risk factors and long-term prognosis for postoperative hypoxemia in patients with acute type A aortic dissection: A retrospective observational study

**DOI:** 10.1097/MD.0000000000032337

**Published:** 2022-12-16

**Authors:** Xiao-Chun Song, Shuai Nie, Ji-Lai Xiao, Xiao Shen, Liang Hong, Shang-Yu Chen, Cui Zhang, Xin-Wei Mu

**Affiliations:** a Cardiovascular Intensive Care Unit (CVICU), Department of Critical Care Medicine, Nanjing First Hospital, Nanjing Medical University, Nanjing, People’s Republic of China.

**Keywords:** acute type A aortic dissection, long-term prognosis, management strategy, postoperative hypoxemia, risk factors

## Abstract

Hypoxemia is 1 of the most common complications in the patients with acute Type A aortic dissection (ATAAD). This study aimed to summarize the risk factors, management strategies and long-term prognosis for postoperative hypoxemia in ATAAD patients. Baseline characteristics and clinical data of all the patients were collected. Patients were divided into 2 groups according to the PaO_2_/FiO_2_ after surgery: Hypoxemia group (n = 142) and Non-hypoxemia group (n = 68). The differences in gender, age, body mass index, operation time, cardiopulmonary bypass (CPB) time, aortic cross-clamping time, deep hypothermic circulatory arrest time, preoperative PaO_2_/FiO_2_, postoperative PaO_2_/FiO_2_, PaO_2_/FiO_2_ before extubating, time of mechanical ventilation, length of intensive care unit stay, length of hospital stay, in-hospital mortality, and overall mortality were compared between the 2 groups. The incidence of postoperative hypoxemia in this study was 67.6% (142/210). body mass index (26.4 ± 3.8 vs 24.4 ± 3.3kg/m^2^, *P* < .001) in the hypoxemia group were markedly higher and CPB time (196.3 ± 41.0 vs 181.0 ± 37.3 minutes, *P* = .010) in the hypoxemia group were significantly longer than those in the non-hypoxemia group. While preoperative PaO_2_/FiO_2_ (229.7 ± 91.4 vs 299.7 ± 101.2mmHg, *P* < .001) was significantly lower than those in the non-hypoxemia group. In the hypoxemia group, PaO_2_/FiO_2_ before extubating was significantly higher than that after operation, and the difference was significant. Logistic regression analysis showed that overweight (odds ratio [OR]: 1.113, *P* = .030), CPB time (OR: 1.009, *P* = .043) and preoperative PaO_2_/FiO_2_ (OR: 0.994, *P* = .001) were independent risk factors for postoperative hypoxemia. Further follow-up results showed no significant difference in long-term mortality between the 2 groups. Logistic regression analysis revealed that PaO_2_/FiO_2_ before extubating (OR: 0.985, *P* < .001), paraplegia (OR: 10.994, *P* = .019), acute renal failure (OR: 12.590, *P* < .001), re-operation (OR: 4.721, *P* = .014) and re-admission to intensive care unit (OR: 13.727, *P* = .001) were independent risk factors for long-term mortality. Our results showed that overweight and prolonged CPB time were risk factors for postoperative hypoxemia in ATAAD patients. While PaO_2_/FiO_2_ before extubating were independent risk factors for long-term mortality, indicating that active correction of hypoxemia and maintain a higher PaO_2_/FiO_2_ before extubating may help to improve the prognosis of the ATAAD patients.

## 1. Introduction

Acute type A aortic dissection (ATAAD) is a cardiovascular emergency with an overall mortality up to 73%. Early diagnosis and prompt surgical intervention are the most effective therapies at present.^[1,2]^ Surgical treatment can save the patient’s life by replacing damaged blood vessels, sealing the endarterial rupture, expanding the true lumen and promoting pseudolumen thrombus formation and mechanization. However, cardiopulmonary bypass (CPB) is required during the operation with extensive surgical trauma. In addition, the replacement of the aortic arch should be carried out under deep hypothermia circulatory arrest (DHCA) and selective cerebral perfusion. During the period of lower body circulatory arrest, major organs such as heart, lung, spinal cord, kidney, liver, gastrointestinal tract, et cetera, are in a state of ischemia and hypoxia, resulting in reversible or irreversible organ dysfunctions. Of which, postoperative hypoxemia caused by lung injury is 1 of the most common complications after surgery. Postoperative hypoxemia will lead to prolonged mechanical ventilation (MV) and increased mortality of the ATAAD patients.^[3,4]^ Thus, early prevention and effective treatment for postoperative hypoxemia are of great importance in ATAAD patients.

Several studies have investigated the risk factors for postoperative hypoxemia and found that body mass index (BMI), preoperative PaO_2_/FiO_2_ ratio and surgery time might be the independent risk factors for postoperative hypoxemia.^[3,4]^ However, the majority of the studies were focused on the short-term outcomes of the ATAAD patients. The purpose of this study was to investigate the risk factors, management strategies and long-term prognosis of postoperative hypoxemia in 210 ATAAD patients who received emergency surgeries in our center.

## 2. Methods

### 2.1. Subjects

The clinical data of the 210 patients with ATAAD who underwent emergency surgery in Nanjing First Hospital, Nanjing Medical University from January 2016 to December 2018 were retrospectively analyzed, and the survivors were followed up to May 2022. All the patients included in this study met the 2010 ACCF/AHA/AATS/ACR/ASA/SCA/SCAI/SIR/STS/SVM guidelines for the diagnosis and management of thoracic aortic disease.^[5]^ Emergency procedures were performed on all the patients within 2 weeks of onset when they were clearly diagnosed with Stanford type A aortic dissection by the computed tomographic angiography of the thoracic-abdominal aorta. Male were the predominant gender and account for 78.1% (164/210) of the patients. Patients were aged 22 to 83 years old, with an average age of (52.9 ± 11.4) years in this study. The surgery types were determined by the cardiothoracic surgeons based on the preoperative computed tomographic angiography of the thoracic-abdominal aorta and intraoperative transesophageal echocardiography. All the surgical procedures were performed under selective cerebral perfusion with deep hypothermic circulatory arrest (DHCA). The specific surgical types and number of cases were displayed in Table 1. The institutional Ethics Committee of Nanjing First Hospital have approved for the conduction and data collection of this study.

**Table 1:** Surgical types for the ATAAD patients.

**Surgical procedure**	**n (%**)
Ascending aorta replacement with Sun’s procedure (total arch replacement and stented elephant trunk implantation)	103 (49.0%)
Bentall with Sun’s procedure	27 (12.8%)
Ascending aorta replacement with right hemiarch replacement	16 (7.6%)
Partial sinus replacement and ascending aorta replacement with Sun’s procedure	14 (6.7%)
Aortic valve plasty and ascending aorta replacement with Sun’s procedure	11 (5.2%)
CABG and ascending aorta replacement with Sun’s procedure	8 (3.8%)
Wheat and Sun’s procedure	8 (3.8%)
Ascending aorta replacement	6 (2.8%)
Aortic valve plasty + Partial sinus replacement + ascending aorta replacement with Sun’s procedure	4 (1.9%)
CABG + Partial sinus replacement + Ascending aorta replacement with right hemiarch replacement	3 (1.4%)
Aortic valve plasty and Ascending aorta replacement with right hemiarch replacement	2 (1.0%)
Bentall and right hemiarch replacement	2 (1.0%)
Non-coronary sinus replacement and Ascending aorta replacement with right hemiarch replacement	1 (0.5%)
Bentall	1 (0.5%)
Aortic valve replacement + right coronary sinus replacement + ascending aorta replacement with Sun’s procedure	1 (0.5%)
mitral valve replacement + ascending aorta replacement with Sun’s procedure	1 (0.5%)
Bentall + right hemiarch replacement + atrial septal defect repair + tricuspid valve plasty	1 (0.5%)
Bentall + mitral valve replacement + tricuspid valve plasty	1 (0.5%)
Simultaneous diversion operation:	
Ascending aorto-femoral artery bypass	6 (2.8%)
Femoral artery - femoral artery bypass	3 (1.4%)
Left subclavian artery-left vertebral artery bypass	8 (3.8%)

ATAAD = acute type A aortic dissection, CABG = coronary artery bypass grafting.

### 2.2. Diagnostic criteria for hypoxemia

The diagnostic criteria for hypoxemia were according to the Berlin Criteria for acute respiratory distress syndrome and literature studies,^[3,6]^ namely, PaO_2_/FiO_2_ ≤ 200mmHg. The patients were divided into hypoxemia group (PaO_2_/FiO_2_ ≤ 200mmHg) and non-hypoxemia group (PaO_2_/FiO_2 _> 200mmHg) based on the postoperative PaO_2_/FiO_2_.

### 2.3. Surgical procedures

All the surgical procedures were performed under general anesthesia. With the dissociation of the right axillary artery, the chest was exposed through the mid-sternal incision, followed by the establishment of cardiopulmonary bypass through the intubation of the right axillary artery and right atrium. Then the ascending aorta was blocked and after its incision, perfusion of the cold cardioplegic solution was performed through the openings of the left and right coronary arteries. The next steps were proximal surgeries (aortic valvuloplasty/partial aortic sinus replacement/ascending aortic replacement/ Bentall/Wheat) and other combined procedures (Table 1). The cardiopulmonary bypass was stopped when the nasopharyngeal temperature decreased to 24 to 25℃, followed by the blockage of the innominate artery, left common carotid artery and left subclavian artery, as well as the opening of the aorta. The brain was then protected by anterograde cerebral perfusion (5~10mL/kg/minutes) through the right axillary artery. The aortic arch was cut open for exploration. In cases that required Sun’s procedure, the stent elephant trunk was then inserted into the true lumen of the descending aorta for tight suture of the proximal end of the stent elephant trunk (with the descending aorta) with the trunk of the 4 branches aortic graft, followed by perfusion through the 4 branches aortic graft to restore the blood perfusion of the lower body in a timely manner. Afterwards, the left common carotid artery was anastomosed with the 4 branches of the aortic graft, and the left common carotid artery was opened to restore the whole-brain perfusion and start rewarming. Subsequently, the proximal end of the aorta was anastomosed with the proximal end of the trunk of the aortic graft, which was then opened after cardiac de-airing for resumption of the heart beat. Finally, the end-to-end anastomosis of the left subclavian artery and innominate artery with the 4 branches of the aortic graft was completed successively. The aortic graft was wrapped with the residual wall and bovine pericardial patch, and shunted with the right atrium to complete the operation. Concomitant procedures included ascending to femoral artery bypass grafting (6 cases), femoral to femoral artery bypass grafting (3 cases), and left subclavian artery to left vertebral artery bypass grafting (8 cases).

### 2.4. Observational index

The incidence of postoperative hypoxemia was analyzed. The differences of preoperative, intraoperative and postoperative variables including gender, age, hypertension, BMI, operation time, CPB time, aortic cross-clamping time, DHCA time, PaO_2_/FiO_2_ before operation, after operation and before extubating, MV time, lengths of intensive care unit (ICU) stay and hospital stay as well as in-hospital mortality and long-term mortality between hypoxemia group and non-hypoxemia group were compared. As for the patients in the hypoxemia group, the differences of PaO_2_/FiO_2_ after operation and before extubating were also compared. In addition, incidence of postoperative neurological dysfunctions (namely delirium, stroke and paraplegia), acute renal failure, re-operation due to bleeding or cardiac tamponade and re-admission to ICU were also reordered.

### 2.5. Management strategies for postoperative hypoxemia

Our management strategies for patients with postoperative hypoxemia were listed as follows: Reasonable MV treatment: The initial setting was tidal volume 8 to 10ml/kg (ideal body weight), respiratory rate 10 to 12 times/minutes, positive end-expiratory pressure 8 to 15cmH_2_O or titrated by esophageal pressure when necessary, and the plateau pressure lower than 25cmH_2_O to ensure adequate alveolar ventilation and prevent end-expiratory alveolar collapse; Recruitment maneuvers: recruitment maneuver is currently an effective strategy for the treatment of atelectasis after ATAAD. In our center, P/V tool (initial pressure: 5 to 10cmH_2_O, peak pressure: 35 to 40cmH_2_O, pressure change: 3cmH_2_O per second, pause 10seconds at the end of inspiration) of the HAMILTON-G5 mechanical ventilator (Hamilton Medical Inc., Switzerland) or pressure control method was used to perform the recruitment maneuvers. Recruitment maneuver therapy could reinflate the collapsed alveoli, improve the ratio of ventilation/blood flow, and improve oxygenation rapidly and effectively. Then, appropriate positive end-expiratory pressure setting was given to prevent the re-collapse of reopened alveoli^[7]^; Reduce inflammation: intravenous glucocorticoids was given for consecutive 3 days postoperatively to reduce the lung injury caused by inflammatory response, to improve pulmonary capillary permeability, to reduce pulmonary intravascular fluid extravasation, and to reduce pulmonary interstitial edema; Correct hypoalbuminemia: postoperative hypoalbuminemia was a common situation after cardiac surgery and could lead to fluid accumulation in the interstitial spaces. Correction of postoperative hypoalbuminemia could increase plasma colloid osmotic pressure and reduce tissue edema, especially pulmonary edema^[8]^; Promote gastrointestinal motility: postoperative gastrointestinal dysfunction was common in ATAAD patients. Prokinetic agents were routinely administered in ATAAD patients to improve the gastrointestinal function after surgery, which could help to reduce the abdominal pressure and increase thoracic volume, potentially to improve the oxygenation.

### 2.6. Statistical analysis

IBM SPSS 26.0 (IBM software) statistical software was used to perform the data analysis. Normal distribution test was performed for each variable. Continuous data conform to normal distribution were expressed as x ± s. Independent sample *t* test was used for comparison of the variables between the 2 groups, and paired *t* test was used for comparison of the variables within 1 group. Categorical data were presented as numerical values (percentages), and Chi-square test was used for comparison between the 2 groups. Logistic regression analysis was used to evaluate the risk factors for postoperative hypoxemia and long-term mortality, and variables with statistical significance in univariate Logistic regression analysis were further brought to the multivariate Logistic regression analysis. At the same time, the variables with statistical differences in multivariate Logistic regression analysis were further evaluated by receiver operating characteristic curve. Survival was estimated by the Kaplan-Meier method with GraphPad Prism 7.0 (GraphPad Software, California). *P* < .05 was considered statistically significant.

## 3. Results

### 3.1. Comparison of baseline characteristics

The incidence of postoperative hypoxemia was 67.6% (142/210) in all the patients of this study. Comparison of the baseline characteristics of the ATAAD patients were displayed in Table 2. No significant differences were detected in terms of preoperative data including gender, age, and medical history of hypertension between the 2 groups. BMI in the patients of the hypoxemia group was significantly higher than those of the non-hypoxemia group (26.4 ± 3.8kg/m^2^ vs 24.4 ± 3.3kg/m^2^, *P* < .001). While the preoperative PaO_2_/FiO_2_ was significantly lower than that in the non-hypoxemia group (229.7 ± 91.4 mmHg vs 299.7 ± 101.2mmHg, *P* < .001). In terms of intraoperative variables, there were no significant differences in operation time, aortic cross-clamping time and DHCA time between the 2 groups. However, the CPB time of the patients in the hypoxemia group was significantly higher than that in the non-hypoxemia group (196.3 ± 41.0minutes vs 181.0 ± 37.3minutes, *P* = .010). There were no significant differences in ICU stay, hospital stay, in-hospital mortality and long-term mortality between the 2 groups in terms of post-operation characteristics. However, PaO_2_/FiO_2_ after operation (131.0 ± 40.3 mmHg vs 265.6 ± 54.6mmHg, *P* < .001) and before extubating (237.9 ± 63.4 mm Hg vs 270.2 ± 59.3mmHg, *P* = .001) were significantly lower in the hypoxemia group than those in the non-hypoxemia group. Accordingly, the time of MV was significantly longer in the non-hypoxemia group. In addition, PaO_2_/FiO_2_ before extubating was significantly higher than that of after extubating in the hypoxemia group (237.9 ± 63.4 mmHg vs 131.0 ± 40.3mmHg, *P* < .001). A total of 22 patients died during hospitalization, with an in-hospital mortality rate of 10.5%. In total, 7 (31.8%) patients died of septic shock and multiple organ failure (MOF), 5 (22.7%) patients died of acute heart failure, 3 (13.6%) patients died of malignant arrhythmia, 2 (9.1%) patients died of uncontrolled gastrointestinal bleeding and the last 5 died of uncontrolled intra-abdominal bleeding (4.5%), tracheal hemorrhage (4.5%), mediastinal hemorrhage (4.5%), multiple organ failure induced by muscle necrosis (4.5%) and intestinal ischemic necrosis (4.5%), respectively.

**Table 2:** Baseline characteristics of the ATAAD patients in the 2 groups.

	**Hypoxemia（n = 142）**	**Non-hypoxemia（n = 68）**	***P* value**
Pre-operation characteristics			
Male, n (%)	110 (77.5)	54 (79.4)	.750
Age, yrs	53.8 ± 11.1	51.7 ± 11.7	.213
Hypertension, n (%)	123 (86.6)	57 (83.8)	.588
BMI, kg/m^2^	26.4 ± 3.8	24.4 ± 3.3	<.001
Pre-op PaO_2_/FiO_2_, mm Hg	229.7 ± 91.4	299.7 ± 101.2	<.001
In-operation characteristics			
Operation time, h	8.2 ± 1.7	7.9 ± 1.4	.217
CPB time, min	196.3 ± 41.0	181.0 ± 37.3	.010
Aortic cross-clamping time, min	112.6 ± 28.4	107.1 ± 21.3	.162
DHCA time, min	23.7 ± 4.4	23.2 ± 4.5	.427
Post-operation characteristics			
Post-op PaO_2_/FiO_2_, mm Hg	131.0 ± 40.3	265.6 ± 54.6	<.001
PaO_2_/FiO_2_ before extubating, mm Hg	237.9 ± 63.4*	270.2 ± 59.3	.001
MV time, h	75.1 ± 50.8	43.0 ± 33.4	<.001
Neurological dysfunctions, n (%)	42 (29.6)	17 (25.0)	.490
Delirium	34 (23.9)	12 (17.6)	.302
Stroke	9 (6.3)	3 (4.4)	.574
Paraplegia	6 (4.2)	3 (4.4)	.950
Acute renal failure, n (%)	31 (21.8)	6 (8.8)	.021
Re-operation, n (%)	11 (7.7)	12 (17.6)	.032
Re-admission to ICU, n (%)	8 (5.6)	4 (5.9)	.942
Length of ICU stay, d	7.6 ± 7.0	6.4 ± 8.7	.267
Length of hospital stay, d	22.3 ± 10.6	21.8 ± 10.4	.730
Hospital mortality, n (%)	18 (12.7)	4 (5.9)	.133
Long-term mortality, n (%)	32 (22.5)	13 (19.1)	.572

ATAAD = acute type A aortic dissection, BMI = body mass index, CPB = cardiopulmonary bypass, DHCA = deep hypothermic circulatory arrest, ICU = intensive care unit, MV = mechanical ventilation, Pre-op = pre operation.

* *P* < .001 versus post-op PaO2/FiO2.

### 3.2. Risk factors for postoperative hypoxemia

Preoperative and intraoperative variables were included in the Logistic regression analysis to evaluate the risk factors for postoperative hypoxemia (Table 3). Univariate Logistic regression analysis showed that BMI (odds ratio [OR]: 1.183, 95% confidence interval [CI]: 1.077–1.298, *P* < .001), preoperative PaO_2_/FiO_2_ (OR: 0.993, 95% CI: 0.990–0.996, *P* < .001) and CPB time (OR: 1.010, 95% CI: 1.002–1.019, *P* = .011) had good predictive values for postoperative hypoxemia. When these variables were further included in the multivariate Logistic regression equation, the results showed that BMI (OR: 1.113, 95% CI: 1.011–1.226, *P* = .030), preoperative PaO_2_/FiO_2_ (OR: 0.994, 95% CI: 0.991–0.998, *P* = .001) and CPB time (OR: 1.009, 95% CI: 1.000–1.018, *P* = .043) was independent risk factors for postoperative hypoxemia.

**Table 3:** Univariate and multivariate Logistic regression analysis of the risk factors for postoperative hypoxemia.

	**Univariate**		**Multivariate**	
	**OR (95%CI**)	***P* value**	**OR (95%CI**)	***P* value**
Pre-operation characteristics				
Male	0.891 (0.439–1.808)	0.750		
Age	1.017 (0.991–1.044)	0.212		
BMI	1.183 (1.077–1.298)	<0.001	1.113 (1.011–1.226)	.030
Pre-op PaO_2_/FiO_2_	0.993 (0.990–0.996)	<0.001	0.994 (0.991–0.998)	.001
In-operation characteristics				
Operation time	1.125 (0.933–1.357)	0.217		
CPB time	1.010 (1.002–1.019)	0.011	1.009 (1.000–1.018)	.043
Aortic cross-clamping time	1.008 (0.997–1.020)	0.163		
DHCA time	1.028 (0.961–1.100)	0.425		

BMI = body mass index, CI = confidence interval, CPB = cardiopulmonary bypass, DHCA = deep hypothermic circulatory arrest, OR = odds ratio, Pre-op = pre-operation.

BMI, preoperative PaO_2_/FiO_2_ and CPB time were further included in the receiver operating characteristic curve to evaluate their predictive values for postoperative hypoxemia. The results showed that the area under the receiver operating characteristic curve (AUROC) of BMI in predicting postoperative hypoxemia was 0.657 (95%CI: 0.577–0.737, *P* < .001) and the cutoff value was 24 kg/m^2^, with a sensitivity of 71.1% and a specificity of 55.9%. The AUROC of preoperative PaO_2_/FiO_2_ in predicting postoperative hypoxemia was 0.710 (95% CI: 0.636–0.785, *P* < .001) and the cutoff value was 234mmHg, with a sensitivity of 73.5% and a specificity of 63.4%. The AUROC of CPB time in predicting postoperative hypoxemia was 0.604 (95%CI: 0.521–0.686, *P* = .015) with a cutoff value of 162 minutes (sensitivity: 83.8%, specificity: 35.3%).

### 3.3. Follow-up

**Figure 1.**
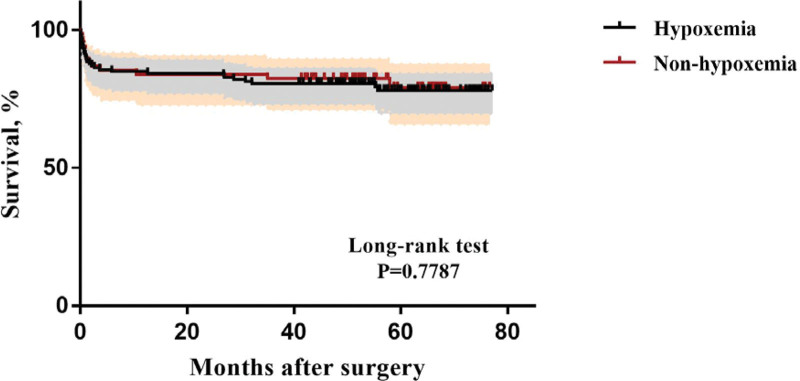
Kaplan-Meier survival curve of the ATAAD patients for overall survival after surgery. ATAAD = acute type A aortic dissection.

22 patients of the 210 patients died during hospitalization (in-hospital mortality: 10.5%). All the discharged patients were followed up to May 2022. A total of 23 patients died during the follow-up, with a total mortality of 21.4% (45/210), including 14 patients in the hypoxemia group and 9 patients in the non-hypoxemia group. Kaplan-Meier survival analysis were displayed in Figure 1. In total, 17 (73.9%) patients died of non-cardiovascular causes, including 8 patients died of recurrence of infection, 6 patients died of other diseases, and 3 died of cerebrovascular accident. Six patients (26.1%) died of cardiovascular causes (3 patients died of unexplained cardiac arrest and the other 3 patients died of acute myocardial infarction, infective endocarditis and abdominal aortic aneurysm rupture, respectively). Four patients received secondary surgical treatment for descending aorta or abdominal aortic dissection (2 patients received total thoracic and abdominal aortic replacement, 1 patient received hybrid surgery, and 1 patient received endovascular aneurysm repair).

The preoperative, intraoperative and postoperative variables were further included in the Logistic regression analysis to assess the risk factors for long-term mortality. The preoperative, intraoperative and postoperative variables with statistically significant differences in univariate analysis were further included in the multivariate Logistic regression equation (Table 4). The results showed that PaO_2_/FiO_2_ before extubating (OR: 0.985, 95% CI: 0.977–0.993, *P* < .001), paraplegia (OR: 10.994, 95% CI: 1.480–81.683, *P* = .019), acute renal failure (OR: 12.590, 95% CI: 4.694–33.769, *P* < .001), re-operation (OR: 4.721, 95% CI: 1.363–16.360, *P* = .014) and re-admission to ICU (OR: 13.727, 95% CI: 2.967–63.498, *P* = .001) were independent risk factors for long-term mortality. The AUROC of PaO_2_/FiO_2_ before extubating in predicting long-term mortality was 0.617 (95% CI: 0.520–0.713, *P* = .017) and the cutoff value was 245mmHg, with a sensitivity of 52.1% and a specificity of 68.9%.

**Table 4:** Univariate and multivariate Logistic regression analysis of the risk factors for long-term mortality.

	**Univariate**		**Multivariate**	
	**OR (95%CI**)	***P* value**	**OR (95%CI**)	***P* value**
Pre-operation characteristics				
Male	1.202 (0.553–2.610)	.642		
Age	1.032 (1.002–1.064)	.037		
BMI	1.089 (1.000–1.187)	.051		
Pre-op PaO_2_/FiO_2_	0.997 (0.994–1.001)	.158		
In-operation characteristics				
Operation time	1.255 (1.024–1.537)	.028		
CPB time	1.012 (1.004–1.020)	.003		
Aortic cross-clamping time	1.015 (1.002–1.027)	.021		
DHCA time	0.941 (0.867–1.023)	.153		
Post-operation characteristics				
Post-op PaO_2_/FiO_2_	0.994 (0.989–0.999)	.020		
PaO_2_/FiO_2_ before extubating	0.991 (0.985–0.997)	.002	0.985 (0.977–0.993)	<.001
MV time	1.004 (0.997–1.010)	.237		
Neurological dysfunctions	2.924 (1.469–5.823)	.002		
Delirium	1.202 (0.553–2.610)	.642		
Stroke	5.895 (1.774–19.589)	.004		
Paraplegia	8.308 (1.989–34.692)	.004	10.994 (1.480–81.683)	.019
Acute renal failure	11.276 (5.062–25.118)	<.001	12.590 (4.694–33.769)	<.001
Re-operation	5.091 (2.069–12.526)	<.001	4.721 (1.363–16.360)	.014
Re-admission to ICU	5.895 (1.774–19.589)	.004	13.727 (2.967–63.498)	.001

BMI = body mass index, CI = confidence interval, CPB = cardiopulmonary bypass, DHCA = deep hypothermic circulatory arrest, ICU = intensive care unit, MV = mechanical ventilation, OR = odds ratio, Post-op = post-operation, Pre-op = pre-operation.

## 4. Discussion

Smoking, preoperative and intraoperative inflammatory response syndrome, perioperative massive blood transfusion, renal insufficiency, obesity, cardiopulmonary bypass, ischemia-reperfusion injury caused by deep hypothermic circulatory arrest and preoperative PaO_2_/FiO_2_ ≤ 300mmHg were previously reported to be risk factors for postoperative hypoxemia in ATAAD patients. Our study further confirmed that overweight, CPB time and preoperative hypoxemia were independent risk factors for postoperative hypoxemia in ATAAD patients. Further follow-up data showed that PaO_2_/FiO_2_ before extubating, paraplegia, acute renal failure, re-operation and re-admission to ICU were independent risk factors for long-term mortality in the ATAAD patients.

According to the definition of obesity by the world health organization, BMI ≥ 25kg/m^2^ is defined as overweight, and BMI ≥ 28kg/m^2^ is defined as obesity. Our study showed that high BMI was an independent risk factor for postoperative hypoxemia in ATAAD patients. Most studies have pointed out that patients with BMI > 25kg/m2 were prone to suffer postoperative hypoxemia.^[9,10]^ In our study, the mean BMI of the patients in the hypoxemia group was 26.4kg/m^2^, which had reached the standard for overweight, and was an important factor leading to postoperative hypoxemia. And the threshold of BMI for predicting postoperative hypoxemia was 24 kg/m^2^, which was consistent with previous studies. CPB induced lung injury has been recognized, and the main mechanisms are systemic inflammatory response syndrome and ischemia-reperfusion injury. In this study, the CPB time in the hypoxemia group was significantly longer than that in the non-hypoxemia group, which was also an important factor leading to postoperative hypoxemia. In this study, patients in the hypoxemia group were overweight and the duration of CPB was prolonged, which contributed to the higher incidence of postoperative hypoxemia in this study than in other studies.

At present, most studies have shown that preoperative PaO_2_/FiO_2_ ≤ 300mmHg was an independent risk factor for hypoxemia after ATAAD surgery.^[3,4,9]^ In our study, the mean preoperative PaO_2_/FiO_2_ was only 229.7mmHg for the ATAAD patients in the hypoxemia group and 299.7mmHg for the ATAAD patients in the non-hypoxemia group, both ≤ 300mmHg. Preoperative hypoxemia was associated with excessive inflammation caused by aortic dissection and would further worsen after surgery. Our results showed that the threshold of preoperative oxygenation index for predicting postoperative hypoxemia was 234mmHg, which was significantly lower than the threshold of previous studies. Unfortunately, the sample size of the non-hypoxemia group in this study was too small, and the results may require further validation in prospective studies with larger sample sizes.

The CPB time of the ATAAD patients is longer than other cardiac patients and also accompanied with deep hypothermic circulatory arrest process, during which the lung is in a complete ischemic-hypoxic state. After blood perfusion is restored, the existing ischemia-reperfusion injury will present aggravation. Pulmonary ischemia-reperfusion injury and systemic inflammatory response caused by CBP can lead to damage to alveolar endothelial cells, epithelial cells and pulmonary vessels, which may cause increased pulmonary capillary permeability, intravascular fluid extravasation, pulmonary interstitial edema, even alveolar edema and respiratory membrane thickening, thus resulting in diffusion dysfunction. Additionally, during CPB and DHCA, the lung is in a non-ventilation state for a long time, and some alveoli collapse and prone to atelectasis and reduced lung volume, which may cause the imbalance of ventilation/blood flow ratio, thereby resulting in the occurrence or aggravation of postoperative hypoxemia.^[11]^ As shown in our study, the postoperative PaO_2_/FiO_2_ decreased significantly when compared with preoperative PaO_2_/FiO_2_ in all the patients, indicating that lung injury was aggravated by the surgical procedure including CPB and DHCA.

According to the potential pathophysiological mechanisms leading to lung injury, our postoperative treatment strategies for patients in the hypoxemia group were as follows: Reasonable treatment measures such as MV, pulmonary recruitment, glucocorticoid therapy, correction of hypoalbuminemia, control of fluid intake, control of ventricular rate and improvement of gastrointestinal motility. Our results showed that those treatment strategies could correct postoperative hypoxemia in most of the ATAAD patients, indicating that our comprehensive treatment has achieved good effects.

Most of the present studies only cared about the in-hospital mortality of the ATAAD patients. We followed up the ATAAD patients for more than 3 years to evaluate their long-term survival in the present study. Our results showed that the long-term mortality for the ATAAD patients was 21.4%. Further regression analysis showed that PaO_2_/FiO_2_ before extubating, paraplegia, acute renal failure, re-operation and re-admission to ICU were independent risk factors for long-term mortality. The cutoff value for PaO_2_/FiO_2_ before extubating in predicting long-term mortality was 245mmHg, suggesting that maintaining a higher oxygenation index before extubating may help to improve the long-term prognosis of the ATAAD patients.

The limitations of this study were as follows: First of all, all the patients received blood gas examinations within half an hour after being transferred to ICU, which may have a certain impact on the postoperative PaO_2_/FiO_2_ results. Secondly, the sample size was relatively small due to the low incidence of ATAAD.

## 5. Conclusions

In conclusion, hypoxemia was 1 of the most common postoperative compilations in the ATAAD patients. Overweight, CPB time and preoperative hypoxemia were independent risk factors for postoperative hypoxemia in ATAAD patients. Additionally, PaO_2_/FiO_2_ before extubating, paraplegia, acute renal failure, re-operation and re-admission to ICU were independent risk factors for long-term mortality. These results indicated that active correction of hypoxemia and maintain a higher PaO_2_/FiO_2_ before extubating may help to improve the prognosis of the ATAAD patients.

## Acknowledgements

The authors gave their appreciation to Dr Shengchen Liu for his contribution in statistical processing in this article.

## Author contributions

**Conceptualization:** Xiao Shen, Cui Zhang, Xin-Wei Mu.

**Data curation:** Xiao-Chun Song, Shuai Nie, Ji-Lai Xiao, Liang Hong, Shang-Yu Chen.

**Formal analysis:** Shang-Yu Chen.

**Funding acquisition:** Xiao Shen.

**Investigation:** Shuai Nie, Ji-Lai Xiao.

**Methodology:** Liang Hong.

**Resources:** Xiao-Chun Song.

**Software:** Liang Hong.

**Supervision:** Cui Zhang.

**Validation:** Ji-Lai Xiao.

**Writing – original draft:** Xiao-Chun Song, Xiao Shen.

**Writing – review & editing:** Cui Zhang, Xin-Wei Mu.

## References

[1] Gudbjartsson T Ahlsson A Geirsson A. Acute type A aortic dissection - a review. Scand Cardiovasc J. 2020;54:1–13. 31542960 10.1080/14017431.2019.1660401

[2] Surgeons TPCoGVoCAoC. Chinese experts, consensus of standardized diagnosis and treatment for aortic dissection. Chin J Thor Cardiovasc Surg. 2017;33:641–54.

[3] Wang Y Xue S Zhu H. Risk factors for postoperative hypoxemia in patients undergoing Stanford A aortic dissection surgery. J Cardiothorac Surg. 2013;8:118. 23631417 10.1186/1749-8090-8-118 PMC3649943

[4] Liu N Zhang W Ma W. Risk factors for hypoxemia following surgical repair of acute type A aortic dissection. Interact Cardiovasc Thorac Surg. 2017;24:251–6. 27756811 10.1093/icvts/ivw272

[5] Hiratzka LF Bakris GL Beckman JA. 2010 ACCF/AHA/AATS/ACR/ASA/SCA/SCAI/SIR/STS/SVM guidelines for the diagnosis and management of patients with thoracic aortic disease: a report of the American college of cardiology foundation/American heart association task force on practice guidelines, American association for thoracic surgery, American college of radiology, American stroke association, society of cardiovascular anesthesiologists, society for cardiovascular angiography and interventions, society of interventional radiology, society of thoracic surgeons, and society for vascular medicine. Circulation. 2010;121:e266–369. 20233780 10.1161/CIR.0b013e3181d4739e

[6] Force ADT Ranieri VM Rubenfeld GD. Acute respiratory distress syndrome: the Berlin definition. JAMA. 2012;307:2526–33. 22797452 10.1001/jama.2012.5669

[7] Guerin C Debord S Leray V. Efficacy and safety of recruitment maneuvers in acute respiratory distress syndrome. Ann Intensive Care. 2011;1:9. 21906333 10.1186/2110-5820-1-9 PMC3224504

[8] Berbel-Franco D Lopez-Delgado JC Putzu A. The influence of postoperative albumin levels on the outcome of cardiac surgery. J Cardiothorac Surg. 2020;15:78. 32393356 10.1186/s13019-020-01133-y PMC7216430

[9] Sheng W Yang HQ Chi YF. Independent risk factors for hypoxemia after surgery for acute aortic dissection. Saudi Med J. 2015;36:940–6. 26219444 10.15537/smj.2015.8.11583 PMC4549590

[10] Gong M Wu Z Xu S. Increased risk for the development of postoperative severe hypoxemia in obese women with acute type a aortic dissection. J Cardiothorac Surg. 2019;14:81. 31023343 10.1186/s13019-019-0888-9 PMC6482483

[11] Pak O Sydykov A Kosanovic D. Lung ischaemia-reperfusion injury: the role of reactive oxygen species. Adv Exp Med Biol. 2017;967:195–225. 29047088 10.1007/978-3-319-63245-2_12

